# The Terpene Synthase Gene Family of Carrot (*Daucus carota* L.): Identification of QTLs and Candidate Genes Associated with Terpenoid Volatile Compounds

**DOI:** 10.3389/fpls.2017.01930

**Published:** 2017-11-09

**Authors:** Jens Keilwagen, Heike Lehnert, Thomas Berner, Holger Budahn, Thomas Nothnagel, Detlef Ulrich, Frank Dunemann

**Affiliations:** ^1^Federal Research Centre for Cultivated Plants, Institute for Biosafety in Plant Biotechnology, Julius Kühn-Institut, Quedlinburg, Germany; ^2^Federal Research Centre for Cultivated Plants, Institute for Breeding Research on Horticultural Crops, Julius Kühn-Institut, Quedlinburg, Germany; ^3^Federal Research Centre for Cultivated Plants, Institute for Ecological Chemistry, Plant Analysis and Stored Product Protection, Julius Kühn-Institut, Quedlinburg, Germany

**Keywords:** *Daucus carota*, terpene synthase (TPS) gene, homology-based gene prediction, monoterpenes, sesquiterpenes, GC-MS, genotyping-by-sequencing (GBS), genome-wide association study (GWAS)

## Abstract

Terpenes are an important group of secondary metabolites in carrots influencing taste and flavor, and some of them might also play a role as bioactive substances with an impact on human physiology and health. Understanding the genetic and molecular basis of terpene synthases (TPS) involved in the biosynthesis of volatile terpenoids will provide insights for improving breeding strategies aimed at quality traits and for developing specific carrot chemotypes possibly useful for pharmaceutical applications. Hence, a combination of terpene metabolite profiling, genotyping-by-sequencing (GBS), and genome-wide association study (GWAS) was used in this work to get insights into the genetic control of terpene biosynthesis in carrots and to identify several TPS candidate genes that might be involved in the production of specific monoterpenes. In a panel of 85 carrot cultivars and accessions, metabolite profiling was used to identify 31 terpenoid volatile organic compounds (VOCs) in carrot leaves and roots, and a GBS approach was used to provide dense genome-wide marker coverage (>168,000 SNPs). Based on this data, a total of 30 quantitative trait loci (QTLs) was identified for 15 terpenoid volatiles. Most QTLs were detected for the monoterpene compounds ocimene, sabinene, β-pinene, borneol and bornyl acetate. We identified four genomic regions on three different carrot chromosomes by GWAS which are both associated with high significance (LOD ≥ 5.91) to distinct monoterpenes and to TPS candidate genes, which have been identified by homology-based gene prediction utilizing RNA-seq data. In total, 65 TPS candidate gene models in carrot were identified and assigned to known plant TPS subfamilies with the exception of TPS-d and TPS-h. TPS-b was identified as largest subfamily with 32 TPS candidate genes.

## Introduction

Terpenoids, also named isoprenoids, are the largest group of plant natural products comprising at least 30,000 different substances and containing a wide assortment of structural types including monoterpenes, sesquiterpenes, diterpenes, and triterpenes (Degenhardt et al., 2009). Terpenoids play a considerable physiological role in the primary metabolism as phytohormones (gibberellic acid and abscisic acid), photosynthesis pigments (carotenoids and chlorophylls) and stabilize membrane structural components (sterols). They have important functions for communication and defense of the plants and help to attract pollinators or predators of herbivores (Pichersky and Gershenzon, 2002; Degenhardt et al., 2003). Terpenoid secondary metabolites are abundant in many essential oils (Lawrence, 1992), resins (Martin et al., 2002) and floral scents (Dudareva and Pichersky, 2000; Magnard et al., 2015). Other terpenes are of pharmaceutical relevance including the monoterpene limonene (Crowell and Gould, 1994) and the sesquiterpene lactone thapsigargin, a bioactive compound of the Apiaceae genus *Thapsia* (Drew et al., 2009). Terpenes are also important in determining the quality of food products including the flavor of wine (Styger et al., 2011), fruit crops such as citrus (Maccarone et al., 1998) and strawberry (Aharoni et al., 2004; Ulrich and Olbricht, 2013). A typical characteristic of monoterpenes and sesquiterpenes is their volatility, and therefore they contribute to the typical flavor and aroma of many plant species (Pichersky and Gershenzon, 2002). In carrots they are important for taste and flavor but are also known to influence bitterness (Kramer et al., 2012).

Most sesquiterpene synthases are localized in the cytosol, whereas monoterpene and diterpene synthases are usually acting in the plastids and have an N-terminal plastid transit peptide upstream of the RRX_8_W motif (Williams et al., 1998). Almost all TPSs contain the DDXXD and the NSE/DTE motifs at the C-terminal region for the metal dependent ionization of the prenyl diphosphate substrate that are essential for their catalytic activities (Tholl, 2006). In addition to the wide range of volatile terpenoids formed directly by TPSs, primary terpene skeletons can be modified further by various enzyme classes, such as the cytochrome P450 hydroxylases, dehydrogenases, glycosyl- and methyltransferases thus increasing their volatility and altering their olfactory properties (Lange et al., 2000; Pateraki et al., 2015).

To date, the TPS gene family consists of more than 100 members from a wide range of plant species. The TPS gene family in plants has been divided into eight subfamilies, designated TPS-a to TPS-h, based on sequence properties and functional characteristics (Chen et al., 2011). TPS-a, TPS-b and TPS-g are angiosperm-specific, with TPS-a containing mostly sesquiterpene and diterpene synthases. TPS-b enzymes catalyze the formation of monoterpenes or isoprenes, and the smaller TPS-g clade consists mostly of monoterpene synthases. TPS-d is gymnosperm-specific, and TPS-h is specific to the spikemoss *Selaginella moellendorffii* (Chen et al., 2011). TPS-c is believed to be the ancestral clade, and it contains genes for copalyl diphosphate synthase. TPS-f derived from TPS-e, thus these two subfamilies have been commonly combined to subfamily TPS-e/f which contains ent-kaurene synthases and other diterpene synthases as well as some mono- and sesquiterpene synthases (Chen et al., 2011).

Complete genome sequencing and annotation revealed that the model plant *Arabidopsis thaliana* contains a set of 40 TPS homologs that cluster into five of the subfamilies of the plant TPS family (Aubourg et al., 2002). TPS gene families have also been extensively studied in sorghum (Paterson et al., 2009), grape (Martin et al., 2010), tomato (Falara et al., 2011), poplar (Irmisch et al., 2014), apple (Nieuwenhuizen et al., 2013), and *Eucalyptus* species (Külheim et al., 2015). Compared to other sequenced genomes, *Eucalyptus* has the largest number of putative TPS genes of any sequenced plant, with a total of more than 100 putative TPS genes each in *E. grandis* and *E. globulus* (Külheim et al., 2015). On the other side, in apple (*Malus domestica*) a comparatively small number of only 10 putative TPS genes is currently known and might be sufficient to account for the diversity of terpenes present in vegetative tissues and apple fruit (Nieuwenhuizen et al., 2013). Besides whole genome studies, transcriptome-based analysis of TPS genes has been performed in several non-model plants including the Asteraceae species *Cichorium intybus* (Testone et al., 2016) and *Artemisia annua* (Wang et al., 2009). Transcriptome analysis of the Apiaceae species *Thapsia laciniata* identified 17 unique TPS sequences among the assembled contigs (Drew et al., 2013). In addition, the isolation and transcription analysis of individual TPS genes and/or functional analysis of their enzymes have been reported for several plant species including strawberry (Aharoni et al., 2003), basil (Iijima et al., 2004), grapefruit (Jia et al., 2005), oregano (Crocoll et al., 2010), and cotton (Yang et al., 2013).

Here, we investigate cultivated carrot *(Daucus carota* subsp. *sativus*), which is an outcrossing biannual species with a high degree of heterozygosity. The haploid genome size of carrot has been estimated at 473 Mbp (Arumuganathan and Earle, 1991), which is a similar small size as reported for rice. Molecular studies have confirmed that domesticated carrots have originated from wild populations in Central Asia (Iorizzo et al., 2013), and that the cultivated carrot germplasm can be divided into two distinct groups, the western and the eastern gene pools (Baranski et al., 2012; Grzebelus et al., 2014). Furthermore, in carrot, a fast decay rate of linkage disequilibrium (LD) is expected, due to the high effective recombination rate in outcrossing species (Flint-Garcia et al., 2003; Clotault et al., 2010). LD decay in carrot is rarely described and to our knowledge, no report exists about whole genome LD decay of carrot. Until now, only two reports exist, dealing with detection of LD decay within carrot genes. In both studies, it was not possible to detect decay of LD within a 4,234 bp-sequence of the CRTISO gene (Soufflet-Freslon et al., 2013) or within intervals of 700–1,000 bp of some carotenoid genes (Clotault et al., 2010).

Carrot is one of the most important root vegetable crops grown worldwide, which has gained popularity in recent decades due to increased awareness of its nutritional value. Carrots do not only provide basic nutrition but do supply nutrition in the form of phytochemicals, such as anthocyanins, phenolic compounds, polyacetylenes, and terpenoids including carotenoids (Arscott and Tanumihardjo, 2010). Many of these compounds contribute to carrot flavor and some of these may contribute as bioactive substances to effects on human physiology and health. Breeding high-quality carrot cultivars would profit from better knowledge of the VOCs involved in taste and aroma. The typical flavor of carrots has been attributed mainly to volatile terpenoids, with mono- and sesquiterpenes representing approximately 98% of the VOCs (Alasalvar et al., 1999; Kjeldsen et al., 2001). Terpenes are generally involved in a harsh or bitter flavor and these flavor attributes were shown to increase directly with terpene content in different carrot genotypes (Kreutzmann et al., 2008; Kramer et al., 2012). Monoterpenes like sabinene and β-myrcene seem to be important contributors to the “carrot top” aroma whereas sesquiterpenes like β-caryophyllene and α-humulene contribute to the “spicy” and “woody” notes (Kjeldsen et al., 2003). Detailed analysis of the terpene profile in leaves and roots revealed differences in total amount and proportions of individual compounds and suggest that terpenoid metabolism differs substantially in these tissues (Habegger and Schnitzler, 2000). A more detailed investigation of carrot root tissues revealed that the biosynthesis of terpenes is mainly localized in the phloem (Hampel et al., 2005). Using headspace solid phase microextraction followed by gas chromatography with flame ionization detection and mass spectrography (HS-SPME-GC-FID and MS), Ulrich et al. (2015) analyzed the diversity of terpene volatile patterns of carrot roots and leaves. In combination with olfactometry, GC led to the identification of substances as “character impact compounds” characterized by flavor dilution factors greater than 1 (Edelenbos et al., 2003). For some compounds also strong correlations of the content in leaves and roots have been shown (Ulrich et al., 2015).

Despite the high importance of mono- and sesquiterpenes for the total volatile profile of carrots, only two recent research papers focused on the identification and functional characterization of two carrot TPS genes called *DcTPS1* and *DcTPS2* (Yahyaa et al., 2015, 2016). Recombinant *DcTPS1* protein produced in an *E. coli*-based expression system mainly the sesquiterpenes (E)-β-caryophyllene and α-humulene, while recombinant *DcTPS2* functioned in *E. coli* as a monoterpene synthase with geraniol as the main product (Yahyaa et al., 2015). Based on the recently published carrot whole genome assembly Iorizzo et al. (2016) performed a preliminary characterization of the carrot TPS family and stated that the *D. carota* genome contains at least 30 TPS genes.

In this paper, we describe a genome wide inventory of the carrot TPS gene family based on the recently published *D. carota* reference genome (Iorizzo et al., 2016) and a homology-based gene prediction approach called GeMoMa (Keilwagen et al., 2016). The contents of volatile terpenoids were semi-quantified by headspace SPME-GC for a collection of carrot accessions representing a broad geographic spectrum of cultivated carrot germplasms. GBS together with GWAS enabled the detection of numerous genomic regions carrying QTLs and TPS candidate genes possibly involved in the genetic control of distinct terpenoid volatiles.

## Materials and methods

### Plant material

Based on results of pre-evaluations a carrot diversity set of 85 cultivars and accessions was composed for a GBS-approach followed by GWAS (Supplementary Table 1). Seeds originating from different gene banks and the JKI working collection were sown and cultivated in 19 cm/30 cm W/H plastic pots in a sand-humus mixture (v/v 3/1) under optimized greenhouse conditions at 25/20°C D/N and 18 h photoperiod. The individual plants were drop irrigated and fertilized each 2-week with 200 mL of a 0.3% Wuxal Super solution (N 8%/P 8%/K 6%; Wilhelm Haug GmbH and Co.KG, Düsseldorf, Germany). Hundred-forty days post-sowing (dps) plants were harvested and each two grams of leaf and root material were frozen for DNA and RNA isolation. Finally root and leaf tissue was shock frozen by liquid nitrogen and stored in a −80°C freezer for volatile analysis.

### Volatile analysis by headspace SPME-gas chromatography

Immediately after thawing the plant material was homogenized for 1 min in a 20% NaCl solution using a Waring blender. For leaves a 10-fold excess (w/v) and for roots a 1.5-fold excess (w/v) of NaCl solution was used. The homogenate was filtered using gauze. For each sample, four 20 mL headspace vials each containing 4 g of solid NaCl for saturation were filled with a 10 mL aliquot of the supernatant and sealed with a magnetic crimp cap including a septum. For automated headspace SPME-GC, a 100 μm polydimethylsiloxane fiber (Supelco, Bellefonte, PA, USA) and a MPS2 autosampler (Gerstel, Mühlheim, Germany) were used. After an equilibration time of 10 min at 35°C under shaking (300 rpm) the fiber was exposed to the headspace for 15 min at 35°C. Desorption was performed within 2 min in the splitless mode and 3 min with split at 250°C. An Agilent Technologies 6890 GC equipped with an HP-5 ms column (0.25 mm i.d., 30 m length, and 0.5 μm film thickness) and FID were used for separation and detection. Carrier gas was hydrogen using a flow rate of 1.1 mL min^−1^. The temperature program was the following: 45°C (5 min), from 45 to 210°C at 3°C min^−1^ and held 25.5 min at 210°C. The volatiles were identified by parallel runs of selected samples on an identically equipped GC-MS by library search (NIST Version 2.0a and MassFinder Version 4), retention indices, and co-elution of authentic samples (except for germacrene). All samples were run with two technical replications.

The commercial software ChromStat2.6 (Analyt, Müllheim, Germany) was used for raw data processing. Data inputs for ChromStat 2.6 were raw data from the percentage reports (retention time/peak area data pairs) performed with the software package Chemstation (version Rev.B.02.01.-SR1 [260]) by Agilent. Using ChromStat2.6, the chromatograms were divided in up to 200 time intervals, each of which represented a peak (substance) occurring in at least one chromatogram of the analysis set. The peak detection threshold was set on the 10-fold value of noise. The values are given as raw data (peak area in counts), which also can be described as relative concentration because of the normalized sample preparation. The semi-quantitation by the software ChromStat 2.6 was focused on 31 VOCs summarized in Table 1.

**Table 1:** Summary of 31 terpenoid volatile compounds which were semi-quantified by HS-SPME-GC-FID.

**No**.	**Substance**	**Abbreviation**	**CAS**	**Substance group**	**Co-el**	**RI**	**Leaves**	**Roots**
1	α-thujene	aTHUJ	2867-05-2	Monoterpene, bicyclic	0	927	1	0
2	α-pinene	aPINE	80-56-8	Monoterpene, bicyclic	1	935	1	1
3	Sabinene	SABI	3387-41-5	Monoterpene, bicyclic	1	975	1	1
4	β-pinene	bPINE	127-91-3	Monoterpene, bicyclic	1	981	1	1
5	β-myrcene	bMYRC	123-35-3	Monoterpene, acyclic	1	991	1	1
6	α-phellandrene	aPHEL	99-83-2	Monoterpene, monocyclic	1	1008	1	1
7	o-cymene	oCYME	527-84-4	Monoterpene, monocyclic	0	1029	1	1
8	Limonene	LIMO	138-86-3	Monoterpene, monocyclic	1	1033	1	1
9	Ocimene	OCIM	13877-91-3	Monoterpene, acyclic	0	1050	1	1
10	γ-terpinene	gTERP	99-85-4	Monoterpene, monocyclic	1	1063	1	1
11	Terpinolene	TERP	586-62-9	Monoterpene, monocyclic	1	1093	1	1
12	Linalool	LINA	78-70-6	Monoterpene alcohol, acyclic	1	1100	1	0
13	Borneol	BORN	507-70-0	Monoterpene, bicyclic	0	1170	1	1
14	Terpinen-4-ol	TERPol	562-74-3	Monoterpene alcohol, monocyclic	1	1181	1	1
15	α-terpineol	aTERP	98-55-5	Monoterpene, monocyclic	0	1193	1	0
16	β-cyclocitral	bCYCL	432-25-7	Monoterpene aldehyde, monocycl.	1	1226	1	1
17	Bornyl acetate	BORNAc	76-49-3	Monoterpene ester, bicyclic	1	1294	1	1
18	δ-elemene	dELEM	20307-84-0	Sesquiterpene, monocyclic	0	1347	1	0
19	α-longipinene	aLONG	5989-08-0	Sesquiterpene, tricyclic	0	1363	1	0
20	α-cubebene	aCUBE	17699-14-8	Sesquiterpene, tricyclic	0	1386	1	0
21	β-caryophyllene	bCARY	87-44-5	Sesquiterpene, bicyclic	1	1435	1	1
22	Geranyl acetone	GERA	3796-70-1	Monoterpene derivate, acyclic	0	1456	0	1
23	β-farnesene	bFARN	502-60-3	Sesquiterpene, acyclic	1	1468	0	1
24	α-caryophyllene	aCARY	6753-98-6	Sesquiterpene, monocyclic	1	1469	1	0
25	Germacrene D	GERM	28387-44-2	Sesquiterpene, monocyclic	1	1496	1	0
26	α-farnesene	aFARN	26560-14-5	Sesquiterpene, acyclic	1	1512	1	0
27	β-bisabolene	bBISA	495-61-4	Sesquiterpene alcohol, monocyclic	0	1516	0	1
28	α-bisabolene	aBISA	17624-44-0	Sesquiterpene, monocyclic	0	1550	1	0
29	Caryophyllene oxide	CARYox	1139-30-6	Sesquiterpene oxide	0	1604	0	1
30	Linalyl isovalerate	LINAis	1118-27-0	Monoterpene ester, acyclic	0	1605	0	1
31	α-bisabolol	aBISol	23089-26-1	Sesquiterpene alcohol, monocyclic	0	1696	1	0
						Sum	26	20
						Cumulative		31
						Mutual		15

*CAS, Chemical Abstracts Service Registry Number; co-el, coelution of authentic reference substances. All compounds were purchased by Sigma-Aldrich Chemie GmbH, Munich, Germany or Carl Roth GmbH, Karlsruhe, Germany*.

### Genotyping-by-sequencing and GWAS

GBS was used for SNP discovery among the 85 carrot accessions listed in Supplementary Table 1. Therefore, total genomic DNA was isolated from 100 mg young leaf material of 85 individual plants of the carrot diversity set using the innuPREP Plant DNA Kit (Analytik Jena, Jena, Germany), quantified by NanoDrop 8000 UV/Vis spectrophotometer (Peqlab, Erlangen, Germany) and sent to LGC Genomics (Berlin, Germany) for library construction and sequencing (150 bp paired-end, Illumina NextSeq). LGC uses an optimized, self-tuning GBS method, called normalized GBS (nGBS) which is based on the restriction enzyme MslI to produce blunt end fragments, and an additional enzyme treatment in a subsequent normalization step. The normalization step is adapted from hybridization kinetics resulting in reduction of abundant fragments. After sequencing, GBS reads were de-multiplexed according to the sample barcodes and sequencing adapter remnants were removed using GBS barcode splitter. Reads were trimmed by base Phred quality (Q score < 20) and reads with a final length shorter than 20 base pairs were discarded before mapping and SNP calling.

We downloaded the carrot reference genome and the genome annotation (Iorizzo et al., 2016) from Phytozome (Goodstein et al., 2012). GBS reads were mapped against the carrot reference genome using BWA-MEM (version: 0.7.7.-r1140) (Li, 2013), and variant calling was performed with samtools (version: 1.2) and bcftools (version: 0.1.19-96b5f2294a) (Li et al., 2009). Subsequently, we filtered the raw variants to obtain high quality bi-allelic SNPs and used Beagle (version: 4.1) (Browning and Browning, 2016) for imputation of missing values. The resulting data set was filtered for markers with minor allele frequency ≥ 5% (MAF) and heterozygosity ≤ 90%. In total, 168,663 bi-allelic, high-quality SNPs were used for further analyses. Next, for each pairwise genotype-genotype combination Rogers' distances were calculated (Reif et al., 2005). Rogers'distance matrix was used to generate a kinship matrix (K-Matrix) by conversion of pairwise distances in a similarity matrix. Based on phenotypic and genotypic data, GWAS was conducted by using the R based software package GAPIT (Lipka et al., 2012; R Core Team, 2014). An association model utilizing the K-matrix as correction for relatedness (K-model, Yu et al., 2006) was fitted for identifying significant marker trait associations. Adapted Bonferroni-Holm correction (Gao et al., 2010; Johnson et al., 2010) (use effective number of independent test instead of number of all tests in the denominator) was used to adjust for multiple testing, as markers in GWAS are not independent due to the assumption of linkage disequilibrium. Effective number of independent tests (principal component cutoff: 0.90) was calculated by using the R based software simpleM (Gao et al., 2008), resulted in a significance threshold of LOD ≥ 5.91. Based on their chromosomal positions significantly associated markers were grouped together. The marker with the highest LOD value was defined as peak marker of a chromosomal region. All significantly associated markers located within an interval of ± 500,000 bp around the peak marker (hereafter: QTL peak marker) were assigned to the same QTL region (Xiao et al., 2016; Cao et al., 2017).

### Discovery of terpene synthase candidate genes and genome reannotation

The homology-based gene prediction program GeMoMa (version: 1.4.2) (Keilwagen et al., 2016) was used to determine potential TPS candidate genes in carrot. Based on the availability of TPS gene models, *Arabidopsis thaliana, Oryza sativa, Populus trichocarpa, Sorghum bicolor* (personal communication with Carsten Külheim, synonyms of *P. trichocarpa* gene names), and *Vitis vinifera* (https://urgi.versailles.inra.fr/content/download/5467/41152/file/VviTPS_20102016_Vitis_12X_2.gff3) were selected as reference organisms yielding in total 334 transcripts in these five organisms. Gene predictions in carrot were made separately for each of the five reference organisms with default parameters except the number of predictions per reference transcript (*p* = 100), experimentally introns and coverage, which have been computed from 20 RNA-seq samples (Iorizzo et al., 2016) that have been mapped to the carrot reference genome using TopHat2 (Kim et al., 2013). The predictions for the five reference organisms were combined using GeMoMa annotation filter (GAF) with default parameters except evidence percentage filter (*e* = 0.1). Finally, these combined predictions were manually filtered obtaining a single high confidence transcript prediction per locus. The final gene annotation, CDS and protein sequences are available in Supplementary Material 1. The Integrative Genomics Viewer (IGV, Robinson et al., 2011) was used as a visualization tool for the interactive exploration of predicted transcripts, annotated *Daucus* genome loci (DCAR sequences, Iorizzo et al., 2016) and EST contigs previously identified as TPS candidates based on *Daucus* transcriptome data (Iorizzo et al., 2011).

As additional evidence, we analyzed the tissue specific gene expression of the final gene predictions. Hence, FeatureCounts (version: 1.5.1) (Liao et al., 2013) was used for determining RNA-seq read counts for the final gene predictions. Subsequently, fragments per kilobase of exon per million fragments mapped (fpkm) values were computed for 20 available RNA-seq samples (Supplementary Table 2). In addition, we used GeMoMa in combination with RNA-seq data for a reannotation of *D. carota* using *A. thaliana* as reference species. Gene predictions were made with default parameters except the experimentally determined introns and coverage.

### RNA isolation and identification of TPS transcripts in roots and leaves

For RNA isolation, frozen leaves and root pieces of cultivars “Blanche” (white root color), “Rotin” (orange), and “Yellowstone” (yellow) were ground to fine powder by using a swing mill. Total RNA was isolated by using the innuPREP Plant RNA Kit (Analytik Jena, Jena, Germany). An additional DNAse digest step (Analytik Jena) was included in this procedure. The qualitatively and quantitatively checked RNA solution was then used to synthesize cDNA with the RevertAid H minus First Strand cDNA Synthesis Kit (Thermo Fisher Scientific, Darmstadt, Germany). PCR primers for semi-quantitative RT-PCR were designed with the webtool Primer3web (Koressaar and Remm, 2007) for TPS candidate genes associated after GWAS with at least one terpene VOC. Additionally, two candidate genes representing each the subfamilies TPS-a and TPS-b, and one representative for TPS-g were selected for the RT-PCR study (for primer sequences, see Supplementary Table 3). With *DcTPS11*, the candidate gene with the highest expression values was included. Based on DNA sequence alignments (ClustalW, DNASTAR Lasergene, Madison, USA) of similar genes located in TPS clusters (i.e., genes on chromosomes 3 and 4) gene-specific primers were designed. Specificity of primers was also tested by a *Local Blast* with the BioEdit software against all 65 *Daucus* TPS coding regions. As reference genes for RT-PCR the constitutive (house-keeping) genes β*-actin* and *elongation factor EF1*α were chosen (Supplementary Table 3). RT-PCR was carried out in a total volume of 20 μL containing 1 μL of the synthesized cDNA solution, 0,4 U of “Phusion” DNA polymerase (Thermo Fisher Scientific), 4 μL of 5x Phusion HF buffer (Thermo Fisher Scientific), 1 μL of each primer (5μM) and 0.2 mM of each dNTP. Amplification conditions were as follows: initial denaturation for 2 min at 98°C; 34 cycles of 98°C for 30 s, 58°C for 30 s, 72°C for 45 s; final extension of 72°C for 5 min. A positive (genomic DNA) and a negative control (water) were included into RT-PCR.

## Results

### Identification, semi-quantitation and genome-wide association of volatiles in leaves and roots

**Figure 1:**
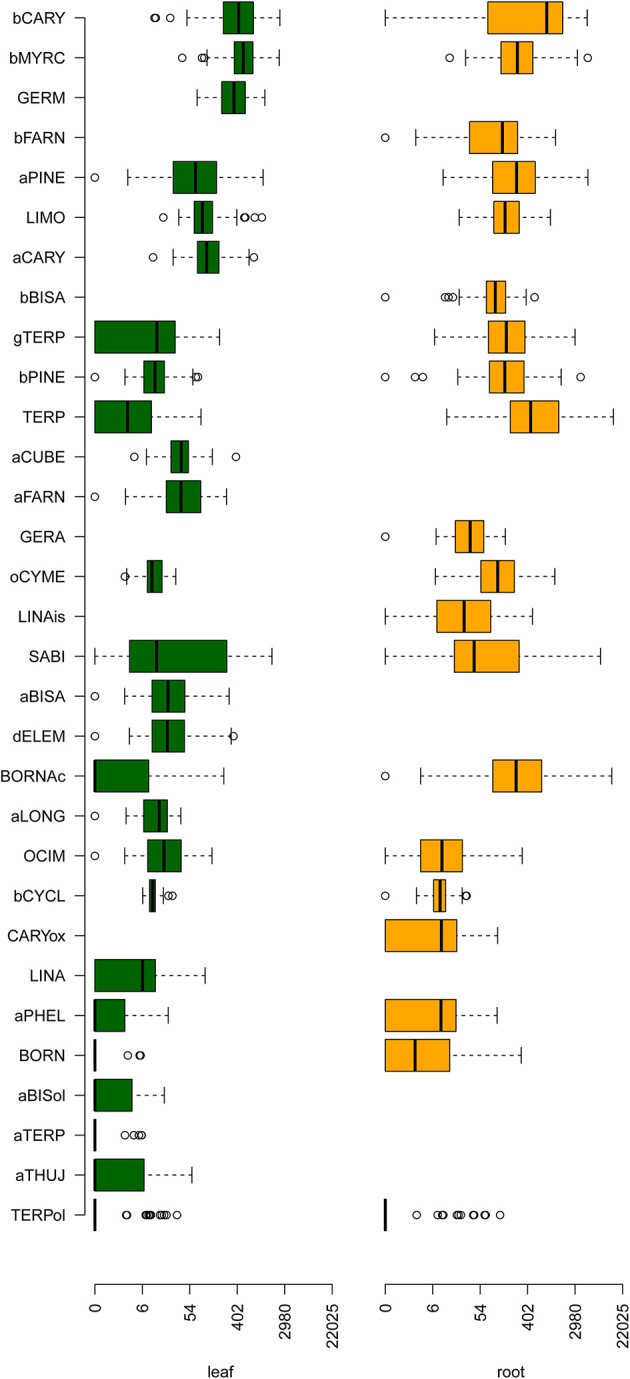
Diversity of terpene contents in leaves and roots of 85 carrot cultivars. The left side of a box displays the 25% quartile, the bold line displays the median, the right side of a box displays the 75% quartile, whiskers indicate the variability outside the 25 and 75% quartile, while dots display outliers. The green boxes on the left side display the terpene contents in leaves, while the orange boxes display the terpene contents in roots. The terpenes are sorted according to the mean of the median content in leaves and roots. The x-axis displaying the terpene content is log1p-transformed. For abbreviated substance names, see Table 1.

The isolation and separation of VOCs from the two plant organs, leaves and roots, were done by headspace SPME-GC. A full multi-compound quantitation using the SPME technique is impossible in complex organic matrices like homogenates of carrot leaves and roots (Schieberle and Molyneux, 2012) and in addition not essential for the aim of this research. Therefore, the data are given as semi-quantitative, relative concentrations (peak areas in counts). The information for phenotyping is contained in the data differences or standardized values for the individual compounds. Table 1 lists the 31 VOCs which were measured either in roots or leaves, or which were identified in both organs. The phenotypic variability of terpene contents among the 85 carrot accessions is shown in Figure 1. The quantitation of the identified terpene volatiles in each carrot accession is presented in Supplementary Table 4. These VOCs belong to the chemical classes of monoterpenes, sesquiterpenes, and several different terpene-derivatives such as terpene alcohols. In leaves 26 out of these 31 VOCs were identified, while only 20 VOCs were identified in roots. Altogether 15 VOCs were identified and semi-quantified in both organs, but also large qualitative differences were found (11 compounds are unique for leaves and also 5 compounds for roots).

The quantitative range of the VOC variation in the carrot collection is tremendous. The mean quantitative level of the data (relative concentrations) differs between leaves and roots as a result of the different sample preparations protocols used (mean value 2,542.79 vs. 6,232.39, Supplementary Table 4). The most abundant compounds in the chromatograms are β-myrcene, β-caryophyllene and germacrene D in leaves and terpinolene, β-caryophyllene and bornyl acetate in roots. Noticeable are the differences between the samples with the maximum and minimum total level of volatiles which are the samples 07 (Purple Haze) and 47 (Nana W 561) (5,930.51 vs. 951.10) in leaves and 55 (Purple Stem Selection) and 50 (Stratova) (28,712.74 vs. 982.35) in roots (Supplementary Table 4). These are factors of 6.2 for leaves and 29.2 for roots, respectively. Supplementary Table 5 shows the results of a correlation analysis of 15 VOCs which are identified in both organs. Five of the compounds (sabinene, β-caryophyllene, ocimene, a-pinene and terpinen-4-ol) show correlations which are significant at a level of *p* < 0.05. This is in accordance with earlier results for sabinene and β-caryophyllene (Ulrich et al., 2015), the substances with the highest correlation between leaves and roots.

Genotyping of the 85 genotypes and data pre-processing resulted in a dataset of 281,394 bi-allelic SNP markers. In total, 112,731 markers were excluded from further analyses, due to minor allele frequency < 5% (106,581 markers) or heterozygosity > 90% (6,150 markers). The remaining 168,663 polymorphic and high-quality markers were used to calculate kinship matrix. Based on these data GWAS was implemented. Compressed mixed linear model approach in GAPIT (Lipka et al., 2012; R Core Team, 2014) with K-matrix as correction for relatedness was conducted and resulted in the identification of 25 and 9 marker trait associations (MTAs) significantly (LOD ≥ 5.91) associated with 11 and 6 VOCs in roots and leaves, respectively (Supplementary Table 6). These significantly associated MTAs were assigned to 21 and 9 QTL regions, respectively (Table 2). The QTL regions are distributed over the whole genome, whereby the majority of QTLs for VOCs were detected on chromosome 7, 8, and 9 in roots and on chromosome 7 in leaves (Table 2). The number of significantly associated markers per QTL region ranged between 1 and 3 (Table 2 and Supplementary Table 6). Most QTLs were detected for the monoterpene compounds ocimene, sabinene, β-pinene, borneol and bornyl acetate. *QTL_r_SABI_4.1* and *QTL_r_TERPol_4*, QTL_r_BORNAc_5.1 and QTL_r_BORN_5.1 or QTL_r_BORNAc_5.2 and QTL_r_BORN_5.2 are located in the same chromosomal region, respectively (Table 2 and Supplementary Table 6). The volatile compounds borneol (BORN) and bornyl acetate (BORNAc) showed significantly associated MTAs for both roots and leaves; however detected QTL regions are not identical. For all other VOCs significantly associated MTAs were only detected either for roots or leaves.

**Table 2:** Quantitative trait loci (QTL) and QTL peak markers significantly associated with volatile compounds (VOC) in roots and leaves (LOD ≥ 5.91).

**Tissue**	**VOC**	**QTL**	**Chromosome**	**Number of markers^a^**	**Position^b^**	**LOD**	**Allelic effect**
Root	aPHEL	*QTL_r_aPHEL_9*	9	1	16,405,410	6.45	−39.31
Root	aPINE	*QTL_r_aPINE_6*	6	3	6,015,083	7.06	−1, 271.08
Root	bBISA	*QTL_r_bBISA_9*	9	1	20,559,777	6.57	−193.84
Root	bMYRC	*QTL_r_bMYRC_7*	7	1	34,705,064	5.91	−1, 103.29
Root	BORN	*QTL_r_BORN_5.1*	5	1	30,630,334	6.18	−52.95
Root	BORN	*QTL_r_BORN_5.2*	5	1	41,039,603	6.87	−60.92
Root	BORNAc	*QTL_r_BORNAc_5.1*	5	1	30,630,334	6.03	−2, 451.47
Root	BORNAc	*QTL_r_BORNAc_5.2*	5	1	41,039,603	6.53	−2, 774.07
Root	bPINE	*QTL_r_bPINE_3*	3	2	271,970	6.05	−737.74
Root	bPINE	*QTL_r_bPINE_7*	7	1	15,005,706	6.26	−816.79
Root	bPINE	*QTL_r_bPINE_8*	8	1	28,567,181	6.64	−851.30
Root	gTERP	*QTL_r_gTERP_8*	8	1	17,369,159	6.03	−647.93
Root	OCIM	*QTL_r_OCIM_1*	1	1	31,849,093	5.97	−66.01
Root	OCIM	*QTL_r_OCIM_3*	3	2	39,077,370	6.66	−63.64
Root	OCIM	*QTL_r_OCIM_8*	8	1	30,233,695	6.06	−51.39
Root	OCIM	*QTL_r_OCIM_9*	9	1	31,325,976	5.96	−59.52
Root	SABI	*QTL_r_SABI_4.1*	4	1	31,232,041	5.95	−2, 276.41
Root	SABI	*QTL_r_SABI_4.2*	4	1	34,821,011	6.09	−2, 056.38
Root	SABI	*QTL_r_SABI_7*	7	1	28,316,072	6.41	−2, 031.26
Root	TERPol	*QTL_r_TERPol_4*	4	1	31,232,041	7.14	−37.80
Root	TERPol	*QTL_r_TERPol_7*	7	1	28,316,072	6.38	−29.89
Leaf	aCUBE	*QTL_l_aCUBE_4*	4	1	3,072,815	5.96	−69.07
Leaf	aTERP	*QTL_l_aTERP_5*	5	1	304,852	6.05	−2.16
Leaf	aTERP	*QTL_l_aTERP_7*	7	1	33,748,718	6.65	−2.40
Leaf	aTHUJ	*QTL_l_aTHUJ_7*	7	1	16,169,936	6.14	−16.30
Leaf	BORN	*QTL_l_BORN_2*	2	1	157,069	5.94	−1.49
Leaf	BORNAc	*QTL_l_BORNAc_2*	2	1	40,042,516	6.02	−49.30
Leaf	BORNAc	*QTL_l_BORNAc_7*	7	1	399,885	6.11	−43.76
Leaf	TERP	*QTL_l_TERP_1*	1	1	6,153,146	5.93	18.93
Leaf	TERP	*QTL_l_TERP_3*	3	1	11,678,474	6.15	−24.76

a *Number of significant associated markers within the QTL region*.

b *Chromosomal position of the QTL peak marker*.

### The TPS gene family in *D. carota*

Bioinformatic searches of the assembled carrot whole genome sequence (Iorizzo et al., 2016) with the homology-based gene prediction program GeMoMa (Keilwagen et al., 2016) and subsequent manual curation and evaluation of transcript predictions identified a total number of 65 TPS candidate gene models in carrot (Table 3, Supplementary Material 1). These 65 TPS gene models do not contain obvious pseudo-genes, since all start with correct start codon, have no frame-shift or internal stop codon, and end with a stop codon. Based on the transcript intron evidence (tie) obtained from GeMoMa using RNA-seq data, the predictions were split in 49 conservative genes with tie ≥ 0.8 and 16 putative genes with tie < 0.8. Comparing the predictions with the annotation (Iorizzo et al., 2016), only 32 have a partial overlap with some annotated transcripts, while 33 do not overlap with any annotation (Table 3). Only 3 out of these 32 overlapping predictions are identical with the annotated transcript (*DcTPS01, DcTPS02, DcTPS29*). The remaining 29 predictions show diverse deviations compared to the annotation—often with additional exons. This might be a reason why these transcripts have not been identified as TPS before.

**Table 3:** List of *Daucus carota* terpene synthase (TPS) gene models sorted by their physical position on the assembled nine carrot chromosomes according to the whole genome sequence (Iorizzo et al., 2016).

**Chromosome^a^**	**Gene name**	**Genomic coordinates**^**a**^			**Locus name^a^**	**EST contig^b^**	**Tie^d^**	**Type^d^**	**TPS**
		**Strand**	**Start**	**Stop**					**Subfamily**
1	*DcTPS32*	−	24861393	24864140	DCAR_002080		1	cons.	TPS-b
1	*DcTPS11*	+	28343809	28346252			1	cons.	TPS-a
1	*DcTPS45*	+	33280212	33282540	DCAR_002829		0.83	cons.	TPS-g
1	*DcTPS46*	+	33285711	33288129	DCAR_002830		0.83	cons.	TPS-g
1	*DcTPS19*	+	33290944	33295685	DCAR_002831		0.83	cons.	TPS-g
1	*DcTPS47*	−	44626531	44658850	DCAR_004091		0.5	put.	TPS-b
1	*DcTPS10*	−	44680158	44682842		ctg6077	1	cons.	TPS-b
1	*DcTPS24*	+	45337090	45339443			1	cons.	TPS-b
1	*DcTPS48*	+	45340690	45343894			1	cons.	TPS-b
1	*DcTPS22*	+	45348547	45351439			1	cons.	TPS-b
1	*DcTPS49*	+	45355908	45363793			0.71	put.	TPS-b
2	*DcTPS41*	−	1270085	1273204			0.83	cons.	TPS-a
2	*DcTPS40*	−	1279452	1282625			1	cons.	TPS-a
2	*DcTPS42*	+	1678080	1680364			1	cons.	TPS-a
2	*DcTPS03*	+	39586545	39589039		ctg1324^c^	1	cons.	TPS-b
3	*DcTPS15*	−	2697938	2700973		ctg15365	1	cons.	TPS-a
3	*DcTPS50*	−	34521894	34524751			0.57	put.	TPS-a
3	*DcTPS37*	−	34584910	34587493			1	cons.	TPS-a
3	*DcTPS08*	−	34633086	34638612			0.83	cons.	TPS-a
3	*DcTPS51*	−	45432441	45435235			0	put.	TPS-b
3	*DcTPS05*	−	45438050	45440460		ctg21245^c^	1	cons.	TPS-b
3	*DcTPS12*	−	45451831	45454062			1	cons.	TPS-b
3	*DcTPS18*	−	45459976	45462408			0.86	cons.	TPS-b
3	*DcTPS25*	+	47468861	47475243	DCAR_012483		1	cons.	TPS-c
3	*DcTPS31*	−	48063948	48068009			1	cons.	TPS-b
3	*DcTPS52*	−	48081099	48084450	DCAR_012537		0.67	put.	TPS-b
3	*DcTPS30*	−	48088855	48092222	DCAR_012538		1	cons.	TPS-b
3	*DcTPS53*	+	48692677	48694890			1	cons.	TPS-a
3	*DcTPS06*	+	48729300	48732085		ctg52846^c^	1	cons.	TPS-a
4	*DcTPS38*	−	15497126	15500214			1	cons.	TPS-a
4	*DcTPS13*	+	25555496	25558124			1	cons.	TPS-a
4	*DcTPS26*	+	31144998	31147390	DCAR_013310		1	cons.	TPS-b
4	*DcTPS04*	−	31217904	31220266	DCAR_013298	ctg13781	1	cons.	TPS-b
4	*DcTPS54*	−	31227164	31230355	DCAR_013297		0.67	put.	TPS-b
4	*DcTPS55*	−	31244459	31247374	DCAR_013294		0.67	put.	TPS-b
4	*DcTPS27*	−	31249549	31251992	DCAR_013293		0.86	cons.	TPS-b
4	*DcTPS09*	−	33893353	33896161	DCAR_012965	ctg260	1	cons.	TPS-b
4	*DcTPS02*^c^	+	33914246	33916610	DCAR_012963	ctg43814^c^	1	cons.	TPS-b
5	*DcTPS56*	+	8252207	8257662	DCAR_016843		0.77	put.	TPS-e
5	*DcTPS28*	+	8266628	8275147	DCAR_016844		0.92	cons.	TPS-e
5	*DcTPS14*	−	20668670	20672094	DCAR_017536	ctg23518	1	cons.	TPS-b
5	*DcTPS17*	+	27521963	27524389	DCAR_018214		1	cons.	TPS-b
5	*DcTPS57*	+	29664194	29669533	DCAR_018422		0.77	put.	TPS-c
5	*DcTPS58*	−	37087498	37090480	DCAR_019208		0.67	put.	TPS-b
5	*DcTPS33*	−	37091126	37102038	DCAR_019208		1	cons.	TPS-b
5	*DcTPS59*	+	39496367	39502226	DCAR_019490		0	put.	TPS-c
6	*DcTPS01*^c^	+	1181665	1185241	DCAR_023152	ctg4929^c^	1	cons.	TPS-a
7	*DcTPS23*	+	18910630	18913238	DCAR_024752		1	cons.	TPS-g
7	*DcTPS60*	+	18916711	18919574	DCAR_024753		0.17	put.	TPS-g
7	*DcTPS61*	−	29317415	29321559			0.33	put.	TPS-a
8	*DcTPS62*	−	14626722	14629935	DCAR_028138		0.67	put.	TPS-b
8	*DcTPS21*	+	14744248	14746910			1	cons.	TPS-b
8	*DcTPS29*	−	17430080	17434674	DCAR_027915		1	cons.	TPS-f
8	*DcTPS44*	−	27097599	27100761	DCAR_026972		1	cons.	TPS-b
8	*DcTPS43*	−	27108437	27111829	DCAR_026971		1	cons.	TPS-b
9	*DcTPS36*	+	8799300	8801682			1	cons.	TPS-a
9	*DcTPS35*	+	8908269	8912243			0.83	cons.	TPS-a
9	*DcTPS63*	+	8935068	8937899			0	put.	TPS-a
9	*DcTPS64*	−	8953248	8958027			0	put.	TPS-a
9	*DcTPS07*	+	8977442	8981304		ctg58617^c^	1	cons.	TPS-a
9	*DcTPS34*	+	8999311	9003484			1	cons.	TPS-a
9	*DcTPS65*	+	23073510	23076811			1	cons.	TPS-a
9	*DcTPS20*	−	31868960	31872669			0.83	cons.	TPS-b
9	*DcTPS39*	−	32232277	32234933	DCAR_031040		0.86	cons.	TPS-a
S3773^e^	*DcTPS16*	+	14141	17230	DCAR_032119		1	cons.	TPS-b

*Transcript intron evidence (tie) was calculated with the GeMoMa software (Keilwagen et al., 2016) using RNA-seq data from Iorizzo et al. (2016). Predictions were split in conservative genes with tie ≥ 0.8 and putative genes with tie < 0.8 and compared with the annotation of Iorizzo et al. (2016) and EST contigs identified previously in the carrot transcriptome (Iorizzo et al., 2011)*.

a *Chromosomes (pseudomolecules), genomic coordinates, orientation and locus names according the carrot whole genome sequence assembly (Iorizzo et al., 2016)*.

b *EST contig no. according the assembled carrot transcriptome (Iorizzo et al., 2011)*.

c *Known carrot TPS genes (DcTPS01, DcTPS02) or predicted full-length carrot TPS cDNA according Yahyaa et al. (2015)*.

d *Tie (transcription intron evidence) according GeMoMa analysis; Type classification: conservative/putative according to tie values (conservative ≥ 0.8)*.

e *Scaffold number according the carrot whole genome sequence assembly (Iorizzo et al., 2016)*.

Utilizing RNA-seq data (Iorizzo et al., 2016), we determined the expression profile of these 65 TPS candidate gene models (Supplementary Table 2) and found diverse patterns of expression within the 20 RNA-seq samples (Supplementary Figure 1). For instance, *DcTPS28* shows stable expression over all 20 samples, whereas *DcTPS03, DcTPS21*, and *DcTPS37* show mainly specific expression for stressed whole storage root, opened whole flowers, and germinating seed at the beginning of germination. *DcTPS11* appears to be the gene with the strongest expression of all carrot TPS candidates. Only two genes (*DcTPS51* and *DcTPS64*) didn't show any transcriptional activity in all 20 transcriptomes.

**Figure 2:**
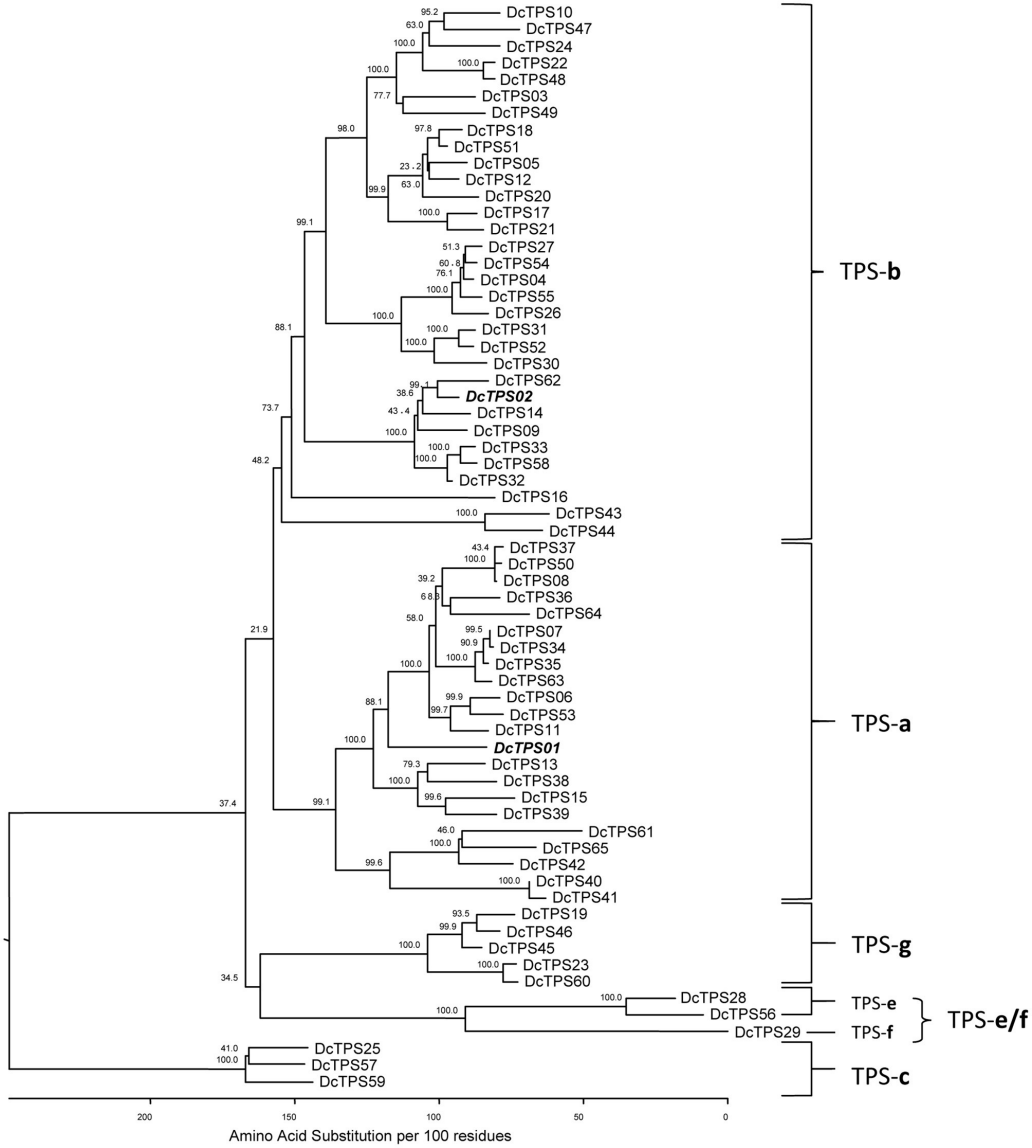
Phylogenetic tree of the deduced *Daucus carota* TPS proteins and their grouping in plant TPS subfamilies. Multiple sequence alignment was performed by ClustalW using the Lasergene (DNASTAR) software package. A phylogenetic tree was constructed using the Kimura distance formula to calculate distance values and bootstrap analysis (1,000 replicates). Numbers indicate bootstrap replication, and branch length is scaled below the tree indicating the number of amino acid substitutions per 100 amino acids. Known genes *DcTPS01* and *DcTPS02* (Yahyaa et al., 2015) are depicted in bold letters.

The 65 TPS candidate genes comprise the previously published sesquiterpene synthase gene *DcTPS1* (*DcTPS01*), the monoterpene synthase gene *DcTPS2* (*DcTPS02*) and four additional full-length EST contigs (Yahyaa et al., 2015). The four ESTs have been preliminary named by their contig number (Yahyaa et al., 2015), however, we propose the gene names *DcTPS03, DcTPS05, DcTPS06, DcTPS07* (Table 3). Based on their sequence similarity to representative TPS from other plant species, the 65 candidate *DcTPS* sequences represent six of the eight plant TPS subfamilies (Table 3, Supplementary Table 7). Phylogenetic analysis of the carrot TPS genes indicates that 22 belong to the TPS-a subfamily which contains predominantly sesquiterpene synthases (Figure 2). A total of 32 genes were found to belong to the TPS-b subfamily of angiosperm monoterpene synthases, and five genes were identified representing the TPS-g subfamily. Another three genes are assigned to subgroup TPS-c, two genes are in TPS-e, and a single TPS gene (*DcTPS29*) was assigned to subfamily TPS-f. The only subgroups with no representative carrot genes were, as expected, the gymnosperm-specific TPS-d subfamily and TPS-h, which is specific to *S. moellendorffii*. The (sub-)classification of DcTPS genes was supported by their alignment with TPS genes identified and characterized in the *Eucalyptus grandis* genome (Külheim et al., 2015) and, concerning the TPSs of subfamilies TPS-c, TPS-e and TPS-f, also with selected TPS genes from *A. thaliana* and *Populus trichocarpa* used also by Külheim et al. (2015) for phylogenetic analyses (Supplementary Figure 2).

The prediction as TPS candidate gene required a TPS open reading frame (ORF) to be of the expected size and that it displays typical structural characteristics such as the intron-exon structure (i.e., generally seven exons for genes of subfamilies TPS-a, TPS-b and TPS-g) and the presence of conserved C-terminal domains such as the Mg^2+^ -binding DDXXD and the NSE/DTE motifs. As shown in Supplementary Table 7, most putative sesquiterpene synthases (TPS-a) have a length of about 540–560 amino acids. More than 20 out of 32 putative monoterpene synthases of subfamily TPS-b are longer than 580 amino acids. This was expected because these proteins are most likely targeted to the plastids due to the presence of a N-terminal transit peptide. All but six of the *Daucus* TPS genes of the subfamilies TPS-a, TPS-b and TPS-g contain seven coding exons (Supplementary Table 7), whereas for the remaining genes 8 exons were predicted. The six genes of the subfamilies TPS-c, TPS-e and TPS-f are characterized by longer sequences and consist of 12–15 exons. Almost all carrot TPS candidate genes showed the sequence motifs characteristic of TPSs, notably the double arginine motif RRX_8_W which is present in most members of subfamilies TPS-a and TPS-b but completely missing in the remaining subfamilies (Supplementary Table 7, Supplementary Figure 3). This motif is known to be involved in producing cyclic monoterpenes and is absent in TPSs that produce acyclic products (Chen et al., 2011). Especially the TPS-g subfamily contains synthases for acyclic monoterpenes known to be involved in floral scent (Dudareva et al., 2003). The three TPS-c genes did also not show the two highly conserved C-terminal motifs (DDXXD, NSE/DTE). However, these motifs appeared to be present in all other TPS subfamilies, with a single exception (*DcTPS64*).

The majority of carrot TPS genes, i.e., the members of subfamilies TPS-a, TPs-b and TPS-g may function as enzymes involved in volatile terpene production. NCBI BlastP searches showed that members of subfamilies TPS-c (*DcTPS25, DcTPS57, DcTPS59*) and TPS-e (*DcTPS28, DcTPS56*) have a predicted putative function as ent-copalyl diphosphate synthase or ent-kaurene synthase and are probably involved in biosynthesis of plant hormone (gibberellin) precursors. The putative function of the single TPS-f gene *DcTPS29* was predicted as a nerolidol-geranyl linalool synthase. When the candidate gene *DcTPS16* was compared with known TPSs of the *Eucalyptus grandis* genome, we found an assignment to the subfamily TPS-b2 (Supplementary Figure 2). TPS-b2 was reported to contain putative isoprene/ocimene synthases (Külheim et al., 2015). The predicted function of *DcTPS16* as a putative isoprene synthase was also indicated by a NCBI BlastP search (data not shown). As demonstrated by Sharkey et al. (2012) isoprene synthase genes form a monophyletic clade of acyclic TPSs in the TPS-b subfamily.

**Figure 3:**
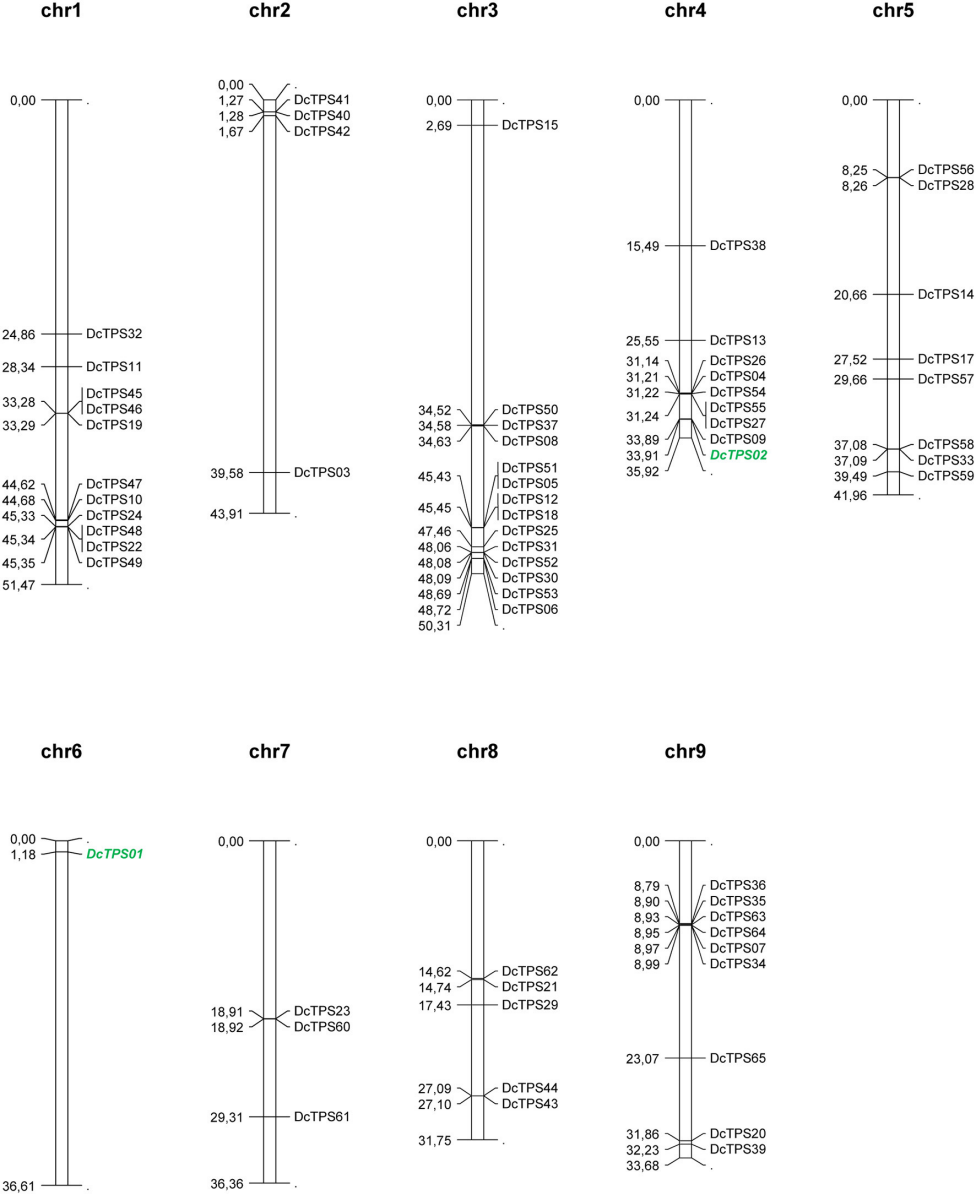
Schematic map presentation of the genomic localization of the 65 carrot TPS genes listed in Table 3. Figures on the left side of the bars show the start position of the CDS of each TPS gene in Mb (mega base pairs). The software *MapChart* 2.2. (Plant Research International, Biometris, Wageningen, Netherlands) was used for map visualization. Known genes *DcTPS01* and *DcTPS02* (Yahyaa et al., 2015) are depicted in green bold letters.

The chromosomal positions of the carrot TPS genes were identified for 64 out of the 65 genes on the nine assembled carrot chromosome sequences (Table 3, Figure 3). Only a single gene (*DcTPS16*) was not linked to the nine carrot chromosomes based on the carrot whole genome sequence. Large differences were found for the number of genes per chromosome. Chromosome 3 comprises 14 TPS genes, whereas chromosome 6 contains a single TPS gene (*DcTPS01*). A majority of the TPS genes were found to co-locate on the same chromosomal genomic region. The largest clusters of TPS sequences were found on chromosome 4 (five genes spanning around 100 kb) and chromosome 9 (six genes spanning about 200 kb, see Table 3, Figure 3). In addition, on chromosome 1 six genes occurred in the region around 45 Mbp. Together with a TPS cluster of 4 genes, on chromosome 3 a total number of 10 TPS genes were located at the end of the assembled pseudomolecule. The large cluster on chromosome 9 contains 6 TPS genes (*DcTPS36, DcTPS35, DcTPS63, DcTPS64, DcTPS07*, and *DcTPS34*) all encoding putative sesquiterpene synthases (TPS-a subfamily). The second-largest cluster on chromosome 4 (with the 5 genes *DcTPS26, DcTPS04, DcTPS54, DcTPS55*, and *DcTPS27*) contains only mono-TPS genes, and the cluster on chromosome 3 (*DcTPS51, DcTPS05, DcTPS12, DcTPS18)* contains also 4 TPS-b genes. The similarity of the sequences of clustered genes is also reflected by their positions in the phylogenetic tree (Figure 2) and suggests the occurrence of multiple gene duplications.

### Candidate gene analysis

Chromosomal start and end positions of the QTL intervals were utilized to compare the QTL regions detected via GWAS in the first subsection with the chromosomal position of TPS candidate genes described in the previous subsection. In total, only 4 out of 30 QTL regions comprise TPS candidate genes. In roots, 6 TPS candidate genes are located in 3 QTL regions, whereas 1 TPS candidate gene is located in 1 QTL region in leaves (Table 4). The number of TPS candidate genes per QTL regions varies between 1 and 5. *QTL_r_SABI_4.1* and *QTL_r_TERPol_4* are located in the same chromosomal region and therefore associated with the same TPS candidate gene cluster (*DcTPS04, DcTPS26, DcTPS27, DcTPS54*, and *DcTPS55*) on chromosome 4.

**Table 4:** Putative TPS candidate genes located in QTL intervals.

**Tissue**	**VOC**	**QTL**	**Chromosome**	**TPS candidate genes**
Root	gTERP	*QTL_r_gTERP_8*	8	*DcTPS29*
Root	SABI	*QTL_r_SABI_4.1*	4	*DcTPS04, DcTPS26, DcTPS27, DcTPS54, DcTPS55*
Root	TERPol	*QTL_r_TERPol_4*	4	*DcTPS04, DcTPS26, DcTPS27, DcTPS54, DcTPS55*
Leaf	BORNAc	*QTL_l_BORNAc_2*	2	*DcTPS03*

*For QTL details (positions, LOD), see Table 2*.

**Figure 4:**
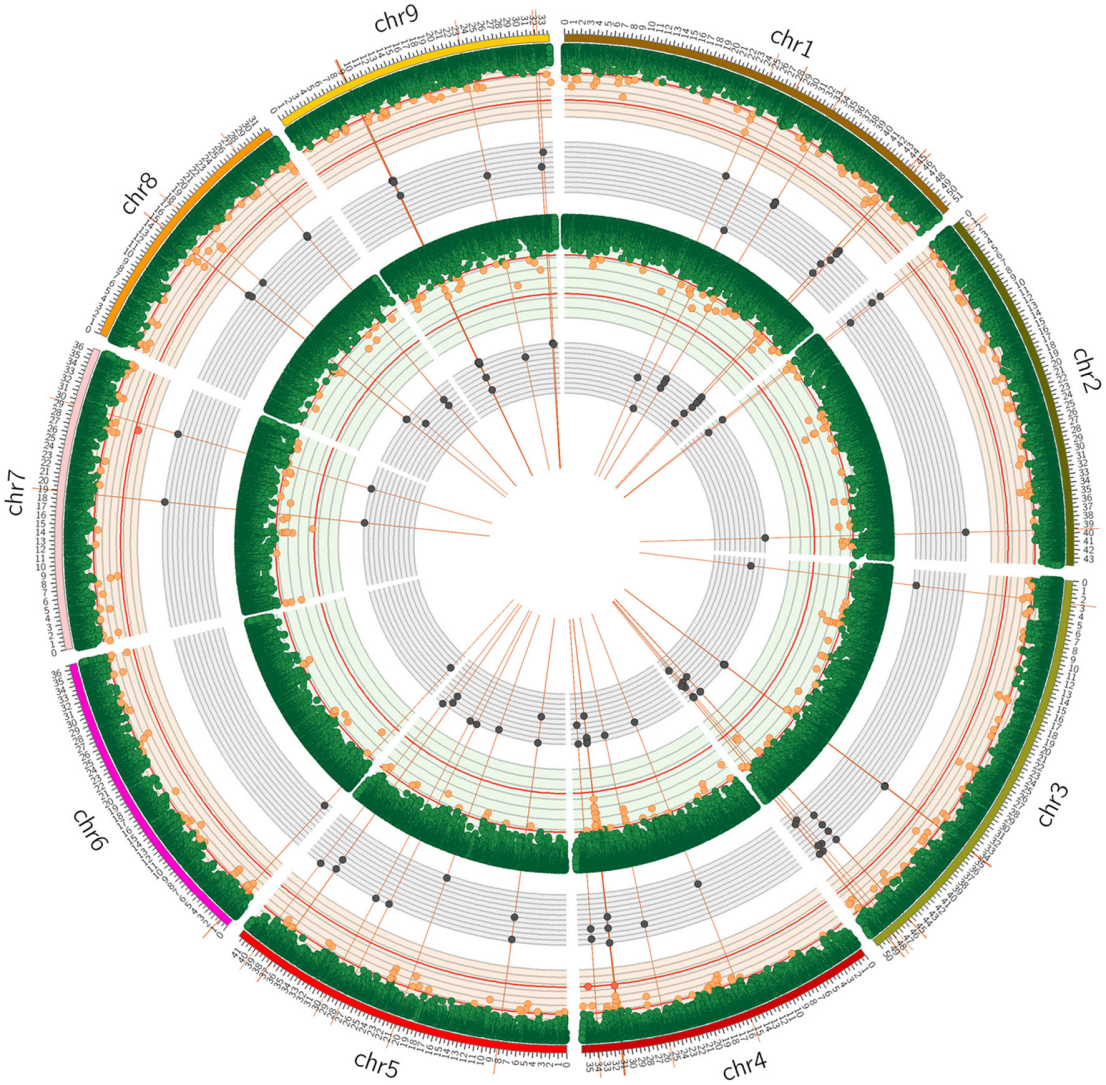
Circos plot for the volatile compound sabinene (SABI). The orange and the green circle represent the results of genome wide association study for SABI in roots and leaves based on 168,663 SNP markers pictured as Manhattan plot. Red horizontal line indicates threshold of significant marker trait associations with LOD ≥ 5.91. Significantly associated marker trait associations highlighted in red. Outer and inner grayish circles show the expression of all carrot TPS candidates in roots and leaves. Vertical red lines indicate the position of the carrot TPS candidates across the genome.

The example of sabinene (SABI) illustrates that it was possible to identify QTL regions which are associated with TPS candidate genes (*QTL_r_SABI_4.1*) as well as QTL regions which carry no TPS candidate genes (*QTL_r_SABI_4.2, QTL_r_SABI_7*) (Figure 4). Hence, Dcarota v2.0 gene annotation (Iorizzo et al., 2016) and the homology-based reannotation (cf. Methods, Supplementary Material 1) were scanned for all genes located within the QTL regions (Supplementary Table 8). Based on this analysis each QTL region comprises at least 9 genes. Focusing on the cytochrome P450 gene family, which is known to modify terpenes, the genes located within the QTL regions were filtered yielding between 0 and 13 cytochrome P450 genes per QTL region (Supplementary Table 8). In total, 14 (Dcarota v2.0 gene annotation) or 13 (homology-based reannotation) out of 30 QTL regions comprise TPS or cytochrome P450 candidate genes, respectively.

To verify predicted gene models and to examine the putative roles of carrot TPS genes in terpene volatile production, we investigated the expression patterns in leaves and roots of three differently colored cultivars. For this approach, specific PCR primers were developed based on the CDS of seven TPS gene models which have been associated by GWAS to QTL regions (Table 4) and for 5 additionally selected TPS candidate genes representing subfamilies TPS-a (*DcTPS11, DcTPS38*), TPS-b (*DcTPS05, DcTPS12*) and TPS-g (*DcTPS60*). As reference genes for semiquantitative RT-PCR, β*-actin* and *elongation factor 1*α (*EF1*α) were used. The totally 12 DcTPS genes were amplified by RT-PCR and studied for differential expression (Supplementary Figure 4). Sequencing of the PCR main products (with the expected fragment size) confirmed the amplification of the right TPS gene (not shown). *DcTPS04, DcTPS05*, and *DcTPS11* seemed to be strongly transcribed in all tested samples. This is in accordance with the expression data, where especially *DcTPS04* and *DcTPS11* showed strong expression in most RNA-seq samples (see Supplementary Table 2). For some genes, such as for instance *DcTPS03* or *DcTPS38* we found some indications, that root tissues have a stronger transcriptional activity than leaves. Interestingly, *DcTPS03* has shown a strong expression in a single transcriptome only (stressed root, Supplementary Table 2) suggesting that this gene might be involved in inducible responses such as resistance to abiotic or biotic stress, but might be stronger expressed due to the general stress induced by the harvest process. TPS genes that were exclusively expressed in leaves or roots were not found. Differences among the cultivars were also less expressed although roots of cv. Blanche showed slightly stronger expression in some genes (*DcTPS27, DcTPS38*). In some cases no transcriptional activity was observed, even after repetition, in a single sample, as for instance for the genes *DcTPS54* (Rotin, leaves) or *DcTPS55* (Rotin, roots).

## Discussion

In the present study, 85 *D. carota* accessions representing the world-wide gene pool of cultivated carrots were analyzed for the qualitative and semi-quantitative composition of VOC patterns of both roots and leaves. From roots, 20 VOCs could be extracted and identified. The majority of these VOCs are monoterpenes, whereas only four sesquiterpenoids were present in the roots, namely β-caryophyllene, β-farnesene, β-bisabolene and caryophyllene oxide. In total, 17 out of 20 VOCs were reported earlier in literature for carrot roots (reviewed in Ulrich et al., 2015). In contrast, the VOCs β-cyclocitral, geranyl acetone and linalyl isovalerate were not yet described in roots. Complementary to the roots, the VOC patterns of carrot leaves were rarely investigated. While Habegger and Schnitzler (2000) investigated the composition of essential oils distilled from leaves, Ulrich et al. (2015) isolated the VOCs from leaves using headspace SPME-GC. Among the 26 compounds measured in the present study in leaves, nine sesquiterpenes or their derivatives were found, but only one of them (β-caryophyllene) was also present in roots. In total, 19 monoterpenes and 12 sesquiterpenes were identified in the present study. Generally, the most abundant compounds in our study are β-myrcene, β-caryophyllene and limonene in leaves, whereas terpinolene, β-caryophyllene and bornyl acetate dominate the VOC patterns of roots. The sesquiterpene β-caryophyllene was also found previously as a main compound in roots, and terpinolene was among the main monoterpenes (Yahyaa et al., 2015). Bornyl acetate, present in our study in high amounts in the roots of nearly all accessions, was found by Yahyaa et al. (2016) to accumulate in high concentration in seeds of some wild carrot relatives. However, despite the comparatively large number of publications dealing with VOCs in carrots, no consensus about typical carrot volatile patterns exists. The lack of agreement in defining the essential VOCs of carrots related to aroma was summarized by Ulrich et al. (2015). Out of the more than 120 compounds which were described in literature, about 80 VOCs are single entries and were identified only in a single study. It cannot be completely ruled out, that in carrot cultivars terpene volatiles playing a decisive role for a typical taste/aroma were lost during the domestication process through an unintentional selection against these VOCs. “Restoring” these missing key terpenes might be reasonable using gene-specific molecular markers, provided that the functional gene(s) controlling these VOCs will be identified. Although the question for the ideal terpenoid VOC composition contributing to a carrot flavor with a high consumer acceptance has still to be answered, the high biological importance of plant terpenes as physiologically active substances involved in plant growth and the interactions of plants with the environment justify further attempts to identify specific TPS genes and to analyze their biochemical functions.

GBS is an ideal platform for studies ranging from single gene markers to whole genome profiling (Poland and Rife, 2012). In carrots, large scale SNP analysis based on the root transcriptome was targeted to an analysis of the effects of domestication on genetic diversity of cultivated carrots (Rong et al., 2014). A GBS approach was used in a further publication to discover SNPs distributed over the whole genome and to evaluate their utility for phylogenetic studies within the genus *Daucus* (Arbizu et al., 2016). To the best of our knowledge, a GBS-based GWAS approach in carrots has not been established until now.

In this work, the combination of a terpene metabolite profiling, GBS and GWAS was used to get insights into the genetic control of terpene biosynthesis and to identify several TPS candidate genes that might be functionally involved in the production of special mono- and sesquiterpenes. A total of 30 QTLs were identified for 6 and 11 terpenoid VOCs measured in carrot leaves and roots. Considering both tissues, most QTLs were detected for the VOCs ocimene (4), sabinene (3), β-pinene (3), borneol (3), and bornyl acetate (3). However, QTLs for bornyl acetate and borneol or sabinene and terpinen-4-ol in roots are located at the same genomic region on chromosome 5 or 4, respectively. This indicates that in these genomic regions common genes might be involved in biosynthesis of different VOCs. However, it has been reported, that terpinen-4-ol can alternatively be produced directly from a non-enzymatic conversion of sabinene, and that borneol can be converted into bornyl acetate (Keszei et al., 2008).

Higher plants generally possess a mid-size TPS gene family resulting from repeated gene duplication, for instance, *A. thaliana* contains 32 putative full length TPSs, rice 34 TPSs, and *Vitis vinifera* 69 TPSs (Chen et al., 2011). In our study, we identified 65 TPS candidate genes in the genome of *D. carota*. It is evident that carrot is among the plants with a high or very high number of TPS genes suggesting a large potential for diversification and variation of terpene metabolism. Amongst sequenced plant species the larger gene families are associated with species that have specialized storage organs for terpenoids such as grape and *Eucalyptus* species (Külheim et al., 2015). Carrot with its typical storage taproot may also belong to this group. From a biochemical point of view, it should be noted, that the number of TPS genes does not necessarily mean a higher diversity of terpenoids. A single terpene synthase has most commonly the ability to form several terpenes from a single substrate. Nearly half of all characterized monoterpene and sesquiterpene synthases have been acknowledged as multi-product enzymes (Degenhardt et al., 2009). Even minor changes in the protein sequence may lead to a neofunctionalization, increasing the diversity of terpenes. For instance, in conifers it was shown, that single or few amino acid substitutions can lead to changes in the product profile of the enzyme (Keeling et al., 2008).

Computationally identified carrot TPS genes fall into all known angiosperm TPS subfamilies, TPS-a to TPS-g, with the exception of the subfamily TPS-d which is gymnosperm-specific (Bohlmann et al., 1998). We identified TPS-b as largest subfamily with a total of 32 potentially functional genes and TPS-a as second largest subfamily with 22 genes. This finding is in contrast to the finding in other plants, as for instance, *A. thaliana*, where 22 of the 32 TPS genes are TPS-a genes (Aubourg et al., 2002), tomato where the majority of TPS genes (12 of the 29 TPS genes) were classified as TPS-a (Falara et al., 2011), and grapevine, which has a substantially extended TPS-a subfamily with 30 TPS genes encoding putative sesquiterpene synthases, compared to a total of 19 genes identified as TPS-b members containing typically monoterpene synthases (Martin et al., 2010). In addition, grapevine was also reported to have an extended TPS-g subfamily. In carrot, we found 5 TPS-g candidates located on only two chromosomes (1 and 7, respectively) which is a higher number than observed in tomato with 2 genes and *A. thaliana* with just a single gene. Furthermore, we found three DcTPS genes of the TPS-c subfamily, two of the TPS-e subfamily and only a single TPS-f gene. The subfamilies TPS-c and TPS-e contain genes which are involved in plant hormone biosynthesis and are not typically represented with multiple gene copies in plant genomes (Bohlmann et al., 1998).

A majority of the carrot TPS genes was found to be genetically linked or even clustered on the nine carrot chromosomes. Especially the TPS gene clusters on chromosomes 1, 3, 4, and 9 might be results of multiple gene duplications. In *A. thaliana*, multiple gene duplication events occurred within the TPS subfamilies TPS-a and TPS-b (Aubourg et al., 2002), and in poplar, the existence of multiple copies of TPS-a and TPS-b genes indicate a similar mechanism of gene duplication (Irmisch et al., 2014). In grape, a large paralogous cluster consisting of 20 complete TPS-a members was found on a single chromosome (Martin et al., 2010). Especially plant genes involved in the plant's defense responses such as TPS genes or genes from the NBS-LRR resistance gene class are known to evolve through gene duplication events (Külheim et al., 2015).

We identified 4 genomic regions on 3 different carrot chromosomes by GWAS which are associated with high significance (LOD ≥ 5.91) to distinct terpene substances and are carrying one or more (clustered) TPS candidate genes. Five candidate genes clustered on chromosome 4 have been classified as genes for monoterpene synthases, which is in correspondence with the products measured (sabinene, terpinen-4-ol). The monoterpene synthase gene *DcTPS03* might be involved in the production of the monoterpene ester bornyl acetate. Recently, it has been noticed that several TPSs are multi-substrate enzymes, capable of synthesizing terpenes of different chain length depending on corresponding substrate availability. Providing alternative substrates *in vitro*, some mono-TPS may also produce sesquiterpenes, whereas some sesqui-TPS are able to produce monoterpenes (Pazouki and Niinemets, 2016). In carrots, recombinant *DcTPS01* protein converted FPP to the predominant sesquiterpene β-caryophyllene, but incubation with GPP led to the production of several monoterpenes (Yahyaa et al., 2015). The monoterpene γ-terpinene was genetically associated with the candidate TPS gene *DcTPS29* of subfamily TPS-f, which was also shown to contain genes with a capacity of multi-substrate use (Pazouki and Niinemets, 2016).

The most significant association (LOD 7.14) was found by GWAS for the substance terpinen-4-ol in roots on the lower part of chromosome 4 containing a cluster of 5 putative mono-TPS genes with high sequence homology. All these genes appeared to be expressed according to the RT-PCR experiment, but presently it is unknown, which gene of this cluster might be the functional gene responsible for the accumulation of terpinen-4-ol in carrot roots. Terpinen-4-ol is known as the main component of the oil of the tea tree *Melaleuca alternifolia* and has a high pharmaceutical importance due to known antibacterial and anti-cancer effects (Calcabrini et al., 2004; Lee et al., 2013). This terpene was measured in roots of only 14 accessions and in leaf tissue of 15 accessions, indicating that this substance was presumably lost in many carrot cultivars during the domestication process. However, the knowledge about the underlying genetics might support breeding efforts with the aim to breed new special carrot types for pharmaceutical use with a high terpinen-4-ol content. An identified QTL for sabinene, a substance involved in “carrot top” aroma (Kjeldsen et al., 2003), was also found to be associated with the gene cluster on chromosome 4. First expression analyses based on selected accessions with very high and low contents of sabinene indicate, that *DcTPS27* might be the decisive gene of the cluster involved in the genetic control of the total level of this important VOC (not shown).

Another interesting result was the finding of *DcTPS11* as the carrot TPS gene with the highest expression, which was confirmed by a strong transcription level as evaluated by RT-PCR. Database searches by NCBI BlastP showed that *DcTPS11* has a predicted function in *Daucus carota* as a sesquiterpene synthase. However, considering all other plant sequences deposited in GenBank, this gene has the highest similarity (with 88% amino acid identity) to the sesquiterpene synthase gene *TgTPS2* from the Apiaceae species *Thapsia garganica*. This gene has been reported to encode a kunzeaol synthase (Pickel et al., 2012). The enzyme is involved in biosynthesis of thapsigargin, a highly bioactive terpenoid compound proposed as a substance to cure prostate cancer (Drew et al., 2009). It would be a challenging future task to reveal the biochemical role and putative importance of *DcTPS11* in carrots by functional studies.

Due to cultivation experience, existing harvest technology as well as due to the high agricultural yields, carrot appears to be a very promising target crop for breeding specific chemotypes with elevated concentrations of specific terpenoids suitable for industrial or pharmaceutical applications. Special carrots with high amounts of bioactive terpenes may also be considered as nutraceuticals that contribute significantly to the known positive effects of carrots on human health. As a first step for such approaches, functional molecular markers might be developed to support the selection of suited breeding genotypes. In future, the biochemical analysis of selected carrot TPS genes, further association analyses targeted to cytochrome P450 genes and other genes modifying the structure of terpenes, and the validation of QTLs in biparental carrot families will contribute to a better understanding of the highly complex biosynthesis of terpenoids in *Daucus*.

## Author contributions

JK, TN, HB, and FD conceived and designed the experimental layout. DU conducted the VOC analysis. TN selected the carrot plant material and helped in interpretation of *Daucus carota* diversity. JK, HL, and TB performed the bioinformatic analysis. FD performed the manual curation and evaluation of transcript predictions and compiled the carrot TPS gene family. JK, HL, DU, and FD drafted the manuscript. JK, HL, HB, TN, DU, and FD contributed to the discussion and interpretation of results and read and approved the final manuscript.

### Conflict of interest statement

The authors declare that the research was conducted in the absence of any commercial or financial relationships that could be construed as a potential conflict of interest.
