# Supplementary material for: The Terpene Synthase Gene Family of Carrot (Daucus carota L.): Identification of QTLs and Candidate Genes Associated with Terpenoid Volatile Compounds
Source: Front Plant Sci. 2017 Nov 9;8:1930. doi: 10.3389/fpls.2017.01930 (PMC5684173; doi:10.3389/fpls.2017.01930)
Supplement: Supplementary file 5 [file Table5.DOCX]

**Supplementary Table 5**

Correlation analysis of 15 VOCs present in leaves and roots. For each VOC, the Pearson correlation was computed between the measurement of the 85 accessions for leaves and roots. Additionally, the p-value was computed using R.

| **substance** | **correlation** | **p-value** |
| --- | --- | --- |
| SABI | 0.72 | 0.0000 |
| bCARY | 0.45 | 0.0000 |
| OCIM | 0.32 | 0.0012 |
| aPINE | 0.27 | 0.0059 |
| TERPol | 0.20 | 0.0364 |
| LIMO | 0.12 | 0.1246 |
| gTERP | 0.12 | 0.1404 |
| bPINE | 0.10 | 0.1785 |
| BORNAc | 0.10 | 0.1822 |
| bMYRC | 0.09 | 0.2031 |
| oCYME | 0.08 | 0.2111 |
| TERP | 0.05 | 0.3205 |
| BORN | 0.02 | 0.4180 |
| aPHEL | -0.01 | 0.5686 |
| bCYCL | -0.11 | 0.8559 |
