# Supplementary material for: The Terpene Synthase Gene Family of Carrot (Daucus carota L.): Identification of QTLs and Candidate Genes Associated with Terpenoid Volatile Compounds
Source: Front Plant Sci. 2017 Nov 9;8:1930. doi: 10.3389/fpls.2017.01930 (PMC5684173; doi:10.3389/fpls.2017.01930)
Supplement: Supplementary file 9 [file Image1.PDF]

Supplementary Figure 1

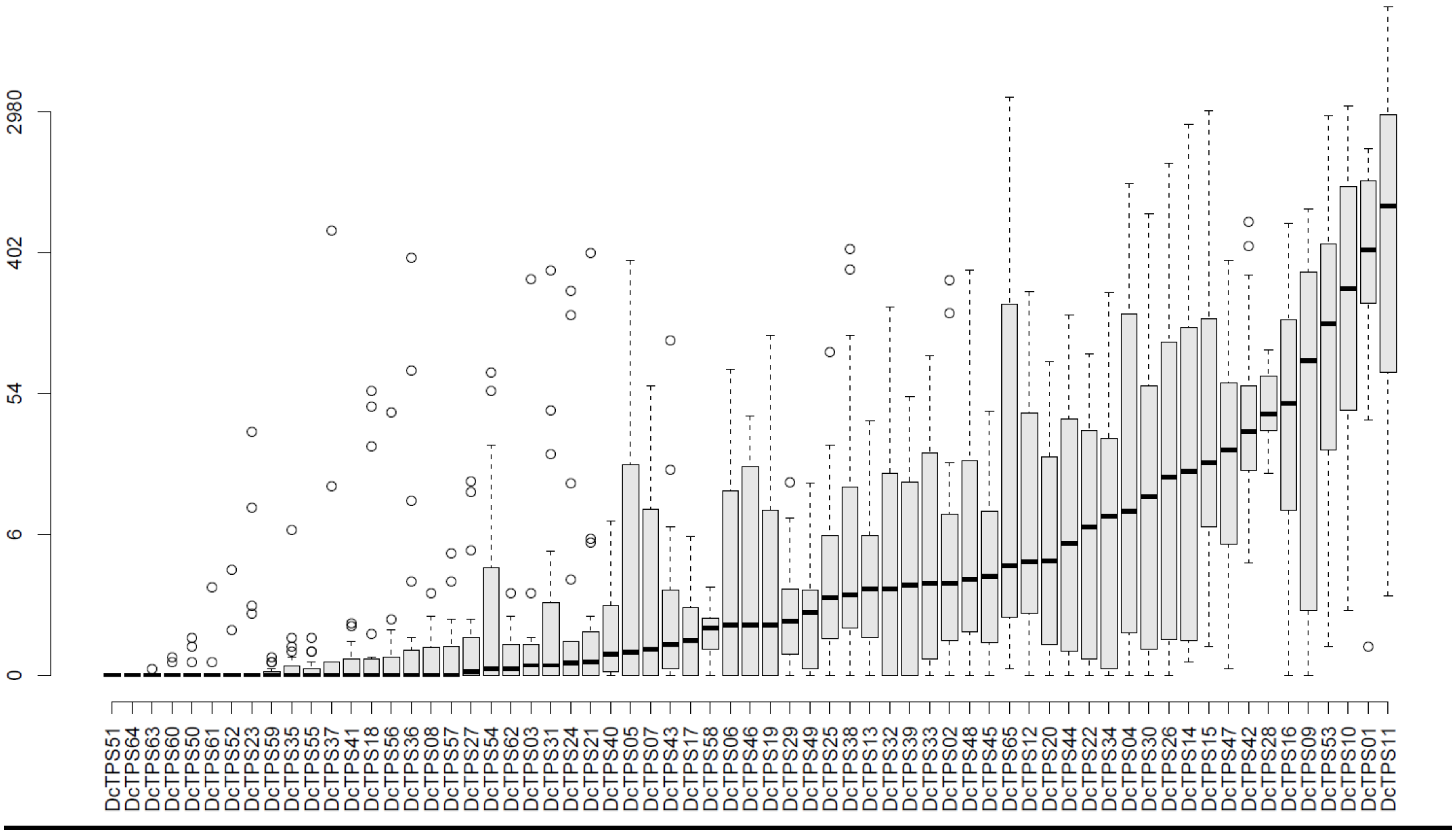

Boxplot of fpkm values for the expression of the 65 TPS candidate genes in 20 RNA-seq samples (Iorizzo et al. 2016). The upper side of a box displays the 25% quartile, the bold line displays the median, the lower side of a box displays the 75% quartile, whiskers indicate the variability outside the 25% and 75% quartile, while dots display outliers. The candidate genes have been sorted according to the median fpkm value.
