# Supplementary material for: The Terpene Synthase Gene Family of Carrot (Daucus carota L.): Identification of QTLs and Candidate Genes Associated with Terpenoid Volatile Compounds
Source: Front Plant Sci. 2017 Nov 9;8:1930. doi: 10.3389/fpls.2017.01930 (PMC5684173; doi:10.3389/fpls.2017.01930)
Supplement: Supplementary file 10 [file Image2.PDF]

Supplementary Figure 2

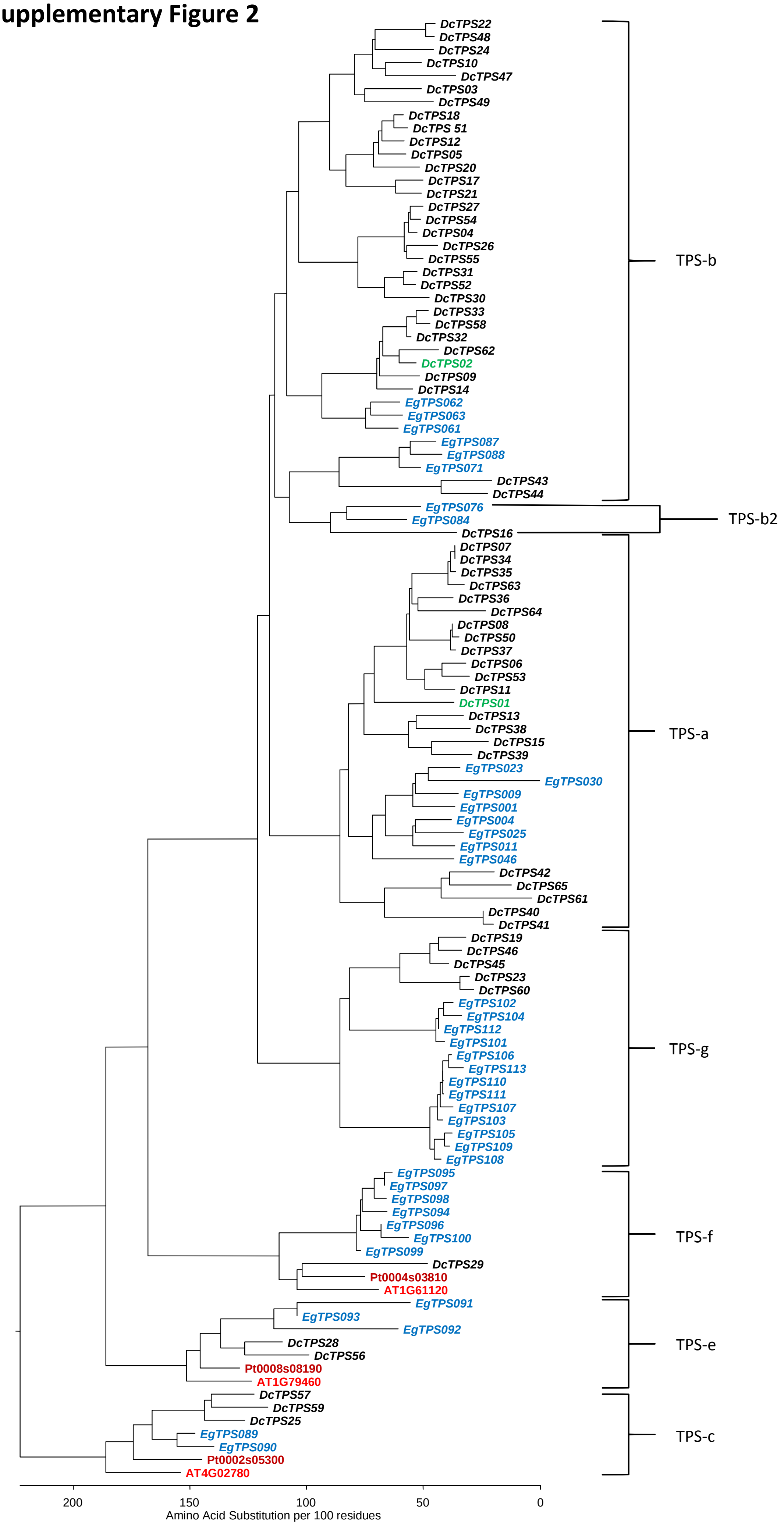

Phylogenetic tree of the deduced *Daucus* TPS proteins (printed in black bold letters) and a selection of plant TPS proteins representing different TPS subfamily members of *Eucalyptus grandis* (blue letters), *Arabidopsis thaliana* (red letters), and *Populus trichocarpa* (dark red letters). All *Eucalyptus*, *Populus* and *Arabidopsis* TPS sequences used for the comparison with *Daucus* TPSs were taken from the work of Külheim et al. (2015). Multiple sequence alignment was performed by ClustalW using the Lasergene (DNASTAR) software. A phylogenetic tree was constructed using the Kimura distance formula to calculate distance values and bootstrap analysis (1,000 replicates). Branch length is scaled below the tree indicating the number of amino acid substitutions per 100 amino acids. Clustering into known plant TPS subfamilies (a-g) is shown.
