# Supplementary material for: The Terpene Synthase Gene Family of Carrot (Daucus carota L.): Identification of QTLs and Candidate Genes Associated with Terpenoid Volatile Compounds
Source: Front Plant Sci. 2017 Nov 9;8:1930. doi: 10.3389/fpls.2017.01930 (PMC5684173; doi:10.3389/fpls.2017.01930)
Supplement: Supplementary file 11 [file Image3.PDF]

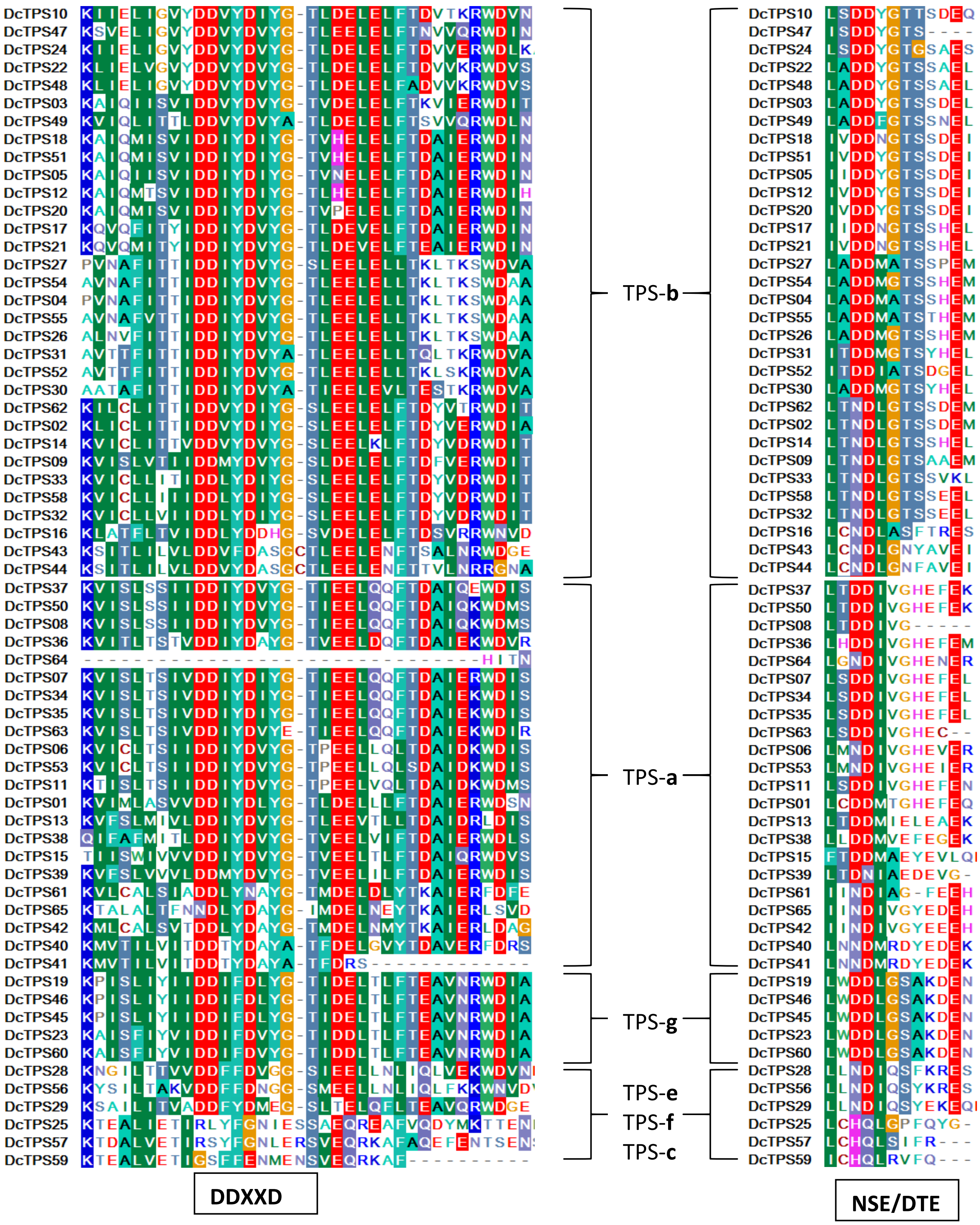

Supplementary Figure 3

Alignment of the conserved C-terminal motifs DDXXD and NSE/DTE within the 65 putative functional *DcTPS* genes identified in the *Daucus carota* genome. Genes are ordered by their position in the carrot TPS dendrogram shown in **Figure 2**.
