# Supplementary material for: The Terpene Synthase Gene Family of Carrot (Daucus carota L.): Identification of QTLs and Candidate Genes Associated with Terpenoid Volatile Compounds
Source: Front Plant Sci. 2017 Nov 9;8:1930. doi: 10.3389/fpls.2017.01930 (PMC5684173; doi:10.3389/fpls.2017.01930)
Supplement: Supplementary file 12 [file Image4.PDF]

Supplementary Figure 4

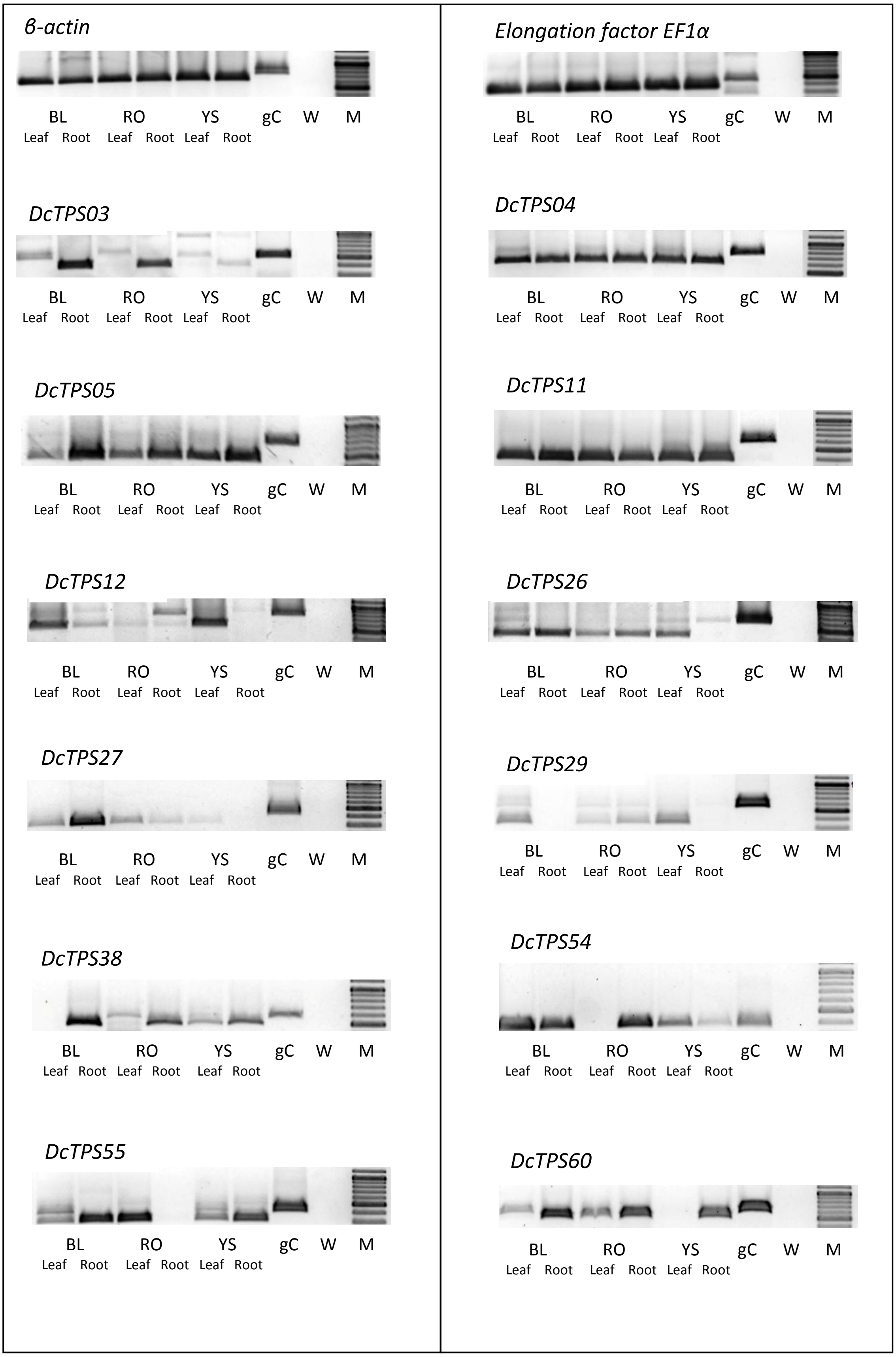

RT-PCR of 12 members of the carrot TPS gene family in leaves and roots of cultivars 'Blanche' (BL), 'Rotin' (RO) and 'Yellowstone' (YS). Positive control of the RT-PCR is carrot (Rotin) genomic DNA (gC), negative control is water (W). As reference genes, the *elongation factor 1α* and *β-actin* were used. M: Genruler (Thermo Fisher) size standard, upper strong band – 1kb, lower strong band – 500 bp
